# End Surface Grinding Machinability of Zirconia Ceramics via Longitudinal–Torsional Coupled Vibration Rotary Ultrasonic Machining

**DOI:** 10.3390/mi16091065

**Published:** 2025-09-21

**Authors:** Fan Chen, Wenbo Bie, Kuohu Li, Xiaosan Ma

**Affiliations:** 1 School of Electrical and Mechanical Engineering, Pingdingshan University, Pingdingshan 467000, China; kuailetianshi028@163.com; 2 School of Mechanical and Power Engineering, Henan Polytechnic University, Jiaozuo 454003, China; 3 School of Intelligent Manufacturing, Henan Polytechnic University, Zhengzhou 450046, China; mmaxiaosan@126.com

**Keywords:** zirconia ceramics, longitudinal–torsional coupled vibration, rotary ultrasonic machining, cutting force, surface roughness

## Abstract

Zirconia (ZrO_2_) ceramics are advanced structural materials that exhibit exceptional performance in aerospace and other heavy-duty applications. Since conventional machining of ZrO_2_ ceramics presents significant challenges, this study employs the longitudinal–torsional coupled rotary ultrasonic machining (LTC-RUM) method for end surface grinding of ZrO_2_ ceramics. To elucidate the material removal mechanism of LTC-RUM, an analysis was conducted from the perspective of individual abrasive grains. Subsequently, LTC-RUM experiments were carried out on ZrO_2_ ceramic samples to investigate the effects of processing parameters on cutting force, surface roughness, and surface morphology. The results show that cutting force decreases with lower spindle speed and ultrasonic power, but increases with higher feed rate and cutting depth. The surface roughness decreases with increasing spindle speed, yet increases with feed rate. Moreover, the surface roughness initially decreases and then increases with increasing ultrasonic power and cutting depth. Compared to conventional machining methods, LTC-RUM significantly reduces cutting force and surface roughness, thereby improving workpiece surface quality. This study provides valuable insights into the application of LTC-RUM for machining ZrO_2_ ceramics and other hard and brittle materials.

## 1. Introduction

Zirconia (ZrO_2_) ceramics combine numerous exceptional physical and chemical properties, such as high hardness, superior strength, remarkable wear resistance, excellent corrosion resistance, and outstanding chemical stability. As a result, they have been widely applied in critical technical fields including aerospace engineering, optoelectronic digital technology, optical glass manufacturing, and medical equipment [1,2]. However, due to their inherent characteristics of high hardness and low fracture toughness [3,4], ZrO_2_ ceramics are prone to surface cracks, subsurface damage, and thermal damage during conventional grinding processes. Consequently, secondary processing is often required to meet practical usage requirements, which increases processing costs and ultimately limits the broader adoption and application of ZrO_2_ ceramics.

Compared to conventional grinding, rotary ultrasonic machining (RUM) combines the material removal of ultrasonic machining and grinding, offering advantages such as enhanced processing efficiency, reduced cutting force, improved surface quality, and minimized tool wear [5,6]. It is particularly suitable for processing hard and brittle materials such as SiCp/Al composites [7], carbon fiber-reinforced composites [8], BK7 [9], and K9 glass [10], as well as sapphire [11]. Pei et al. [12] proposed a model for predicting the material removal rate during RUM, providing valuable insights into this technique. Subsequently, Liu et al. [13] developed a mathematical model assuming that brittle fracture is the primary mechanism for material removal in ultrasonic machining of brittle materials, and they established relationships between machining parameters and cutting forces. Xiao et al. [14], considering the critical cutting depth at the transition stage from brittleness to ductility along with average cutting depth at both ductile and brittle stages, established a cutting force model for RUM side grinding of ZrO_2_ ceramics. Yang et al. [15] introduced a predictive model for cutting forces in RUM of ZrO_2_ ceramics based on single abrasive particle scratch tests, and their findings were experimentally validated. Compared to conventional grinding methods, RUM improved workpiece surface quality while reducing tool wear. Huang et al. [16] developed a theoretical model to predict cutting forces in RUM end grinding of BK7 optical glass, and their study provided an effective approach for selecting process parameters to achieve high efficiency and precision machining. However, the research was based on one-dimensional (1D) RUM generated by ultrasonic-assisted machining (UAM). Although optimizing the 1D RUM scheme enhances its performance, it remains limited by extreme conditions.

Compared to conventional grinding, rotary ultrasonic machining (RUM) integrates the material removal mechanisms of ultrasonic machining and grinding, offering advantages such as higher processing efficiency, reduced cutting forces, improved surface quality, and minimized tool wear [5,6]. It is particularly effective for machining hard and brittle materials, including SiCp/Al composites [7], carbon fiber-reinforced composites [8], BK7 glass [9], K9 glass [10], and sapphire [11]. Pei et al. [12] proposed a model to predict the material removal rate in RUM, contributing valuable insights into the understanding of this technique. Subsequently, Liu et al. [13] developed a mathematical model assuming that brittle fracture is the dominant material removal mechanism during ultrasonic machining of brittle materials, and established relationships between machining parameters and cutting forces. Xiao et al. [14] developed a cutting force model for RUM side grinding of ZrO_2_ ceramics by considering the critical cutting depth at the transition from brittle to ductile behavior, as well as the average cutting depths in both ductile and brittle regimes. Yang et al. [15] introduced a predictive model for cutting forces in RUM of ZrO_2_ ceramics based on single abrasive particle scratch tests, and their results were experimentally validated. Compared to conventional grinding methods, RUM has been shown to improve surface quality while reducing tool wear. Huang et al. [16] proposed a theoretical model to predict cutting forces in RUM end grinding of BK7 optical glass, offering an effective approach for selecting process parameters to achieve high efficiency and precision. However, this research was based on one-dimensional (1D) RUM, which is generated by ultrasonic-assisted machining (UAM). Although optimizing the 1D RUM setup enhances its performance, it remains constrained under extreme conditions.

**Figure 1:**
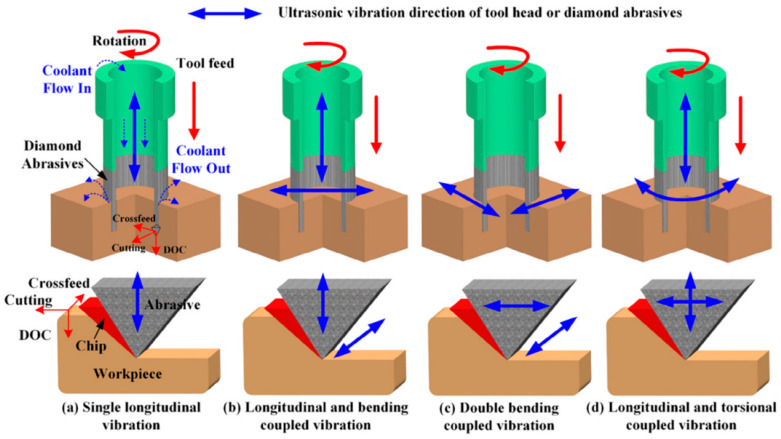
Illustration of different modes of ultrasonic vibrations in VAM [21].

To overcome the limitations of one-dimensional rotary ultrasonic machining (1D RUM), a two-dimensional vibration-assisted machining (2D VAM) technique has been developed and applied in practical machining operations. The 2D VAM method primarily comprises three variants: longitudinal-bending VAM [17], double-bending VAM [18], and longitudinal–torsional VAM [19,20], as illustrated in Figure 1.

Among the three types of ultrasonic vibrations, the latter type, known as longitudinal–torsional coupled vibration, is widely used in various ultrasonic-assisted machining processes due to its simple structure and ease of implementation. Wang et al. [21] reported that LTC-RUM can reduce cutting force by over 50% when processing ceramic matrix composites, as compared to conventional RUM (CON-M). Ma et al. [22] observed a significant improvement in surface quality during their experimental study on the machinability of ZrO_2_ ceramics using LTC-RUM in comparison with CON-M. Qiao et al. [23] proposed a method for simulating cutting force in LTC-RUM face grinding of ceramic matrix composites, achieving a maximum prediction error of 18%. To investigate the influence of process parameters on cutting force, Chen et al. [24] developed a cutting force model for LTC-RUM machining of ZrO2 ceramics, incorporating spindle speed, feed rate, and ultrasonic power, with a maximum prediction error below 16%. Jin et al. [25] compared LTC-RUM and CON-M methods for machining SiC_f_/SiC ceramic matrix composites and found that under LTC-RUM conditions, cutting force was reduced by 64.4% and surface roughness by 44.5%. Despite the accurate assessment of cutting force in LTC-RUM, further clarification is required regarding the material removal mechanism under longitudinal–torsional coupled vibration. Su et al. [26] investigated the material removal mechanism and surface integrity of SiC_f_/SiC composites using longitudinal–torsional ultrasonic vibration-assisted grinding (LTUAG) and conventional grinding (CG). In comparison to CG, LTUAG decreases fiber pull-out, suppresses crack propagation between fibers, and enhances the percentage of ductile material removal. The peak reductions in normal and tangential forces achieved by LTUAG relative to CG are approximately 40% and 47.7%, respectively, with surface roughness being reduced by up to 12.8%. Zhang et al. [27] examined rotary longitudinal–torsional ultrasonic machining (RTLUM) applied to unidirectional carbon fiber-reinforced polymer (CFRP). Their findings revealed that RTLUM facilitates the automatic discharge of rod-shaped chips while mitigating tool-chip adhesion and minimizing heat accumulation and tool degradation. Moreover, the influence of cutting depth and other machining variables, including spindle speed, feed rate, and ultrasonic amplitude, on cutting force during RUM end face grinding has yet to be fully understood.

Although there is growing research on LTC-RUM, limited studies have specifically examined its application in end surface grinding. To fill this knowledge gap, this research focuses on the analysis of cutting force and surface roughness in both LTC-RUM and conventional machining methods. Initially, the material removal mechanism is examined at the level of individual abrasive grains. Following this, experimental tests are carried out to evaluate and compare cutting force, surface roughness, and surface topography against those achieved with CON-M.

## 2. Mechanism of Material Removal

### 2.1. Kinematic Analysis of a Single Abrasive Grain

**Figure 2:**
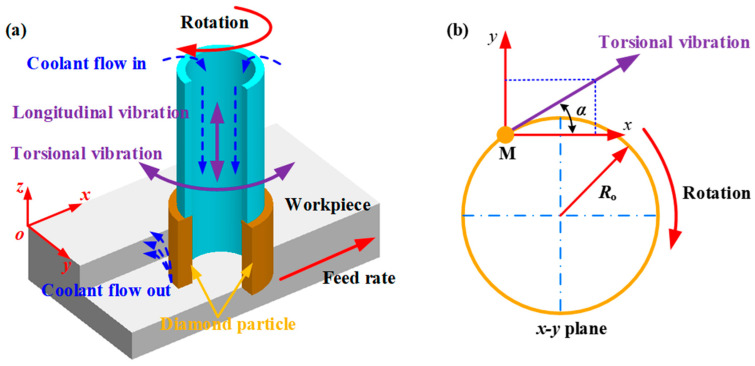
LTC-RUM end surface grinding scheme (**a**) and kinematics (**b**).

The schematic representation of LTC-RUM end surface grinding is illustrated in Figure 2.

During the machining process, three distinct motions occur, as shown in Figure 2a: the electroplated diamond core drill rotates at a spindle speed *n* while undergoing simultaneous vibrations in both the longitudinal (*A*_L_) and torsional (*A*_T_) directions, and the workpiece advances at a feed rate of *f_r_*. By applying the principle of motion independence within the *xy*-plane, the torsional vibration in the circumferential direction can be resolved into two orthogonal components—one along the *x*-axis and the other along the *y*-axis—as illustrated in Figure 2b.

**Figure 3:**
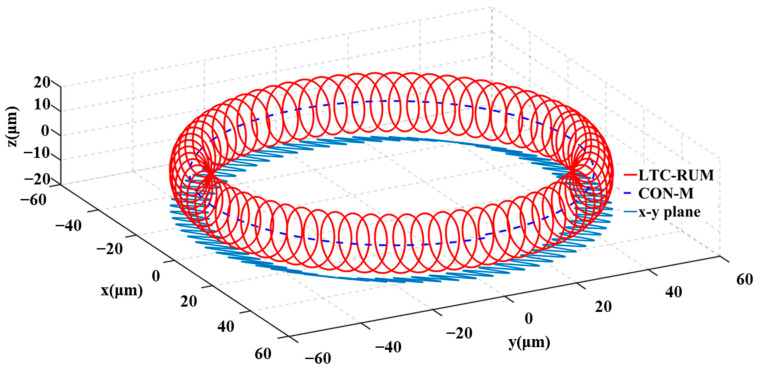
Motion paths of individual abrasive grains in LTC-RUM and CON-M grinding modes.

In the LTC-RUM end face grinding process, the abrasive particle experiences longitudinal and torsional vibrations that share the same frequency and phase properties. Additionally, the arc length corresponding to the rotational angle is approximately equal to the torsional amplitude. As a result, the trajectory of a single abrasive grain can be described by the following expression:

(1) $$\left\{\begin{array}{l} x=R_{o}\sin\left(\frac{\pi nt}{30}\right)+A_{T}\sin\left(2\pi ft\right)\cos\alpha +f_{r}t\\ y=R_{o}\sin\left(\frac{\pi nt}{30}\right)+A_{T}\sin\left(2\pi ft\right)\sin\alpha \\ z=A_{L}\sin\left(2\pi ft+\theta \right)+a_{p} \end{array}\right.$$

where *R*_o_ signifies the tool’s outer radius, *θ* represents the phase shift between longitudinal and torsional oscillations, and α refers to the angle between the torsional vibration component and the circumferential direction (with α ∈ [0, 2π]). Meanwhile, *A*_L_ and *A*_T_ correspond to the amplitudes of the longitudinal and torsional vibrations, respectively. Based on Equation (1), the trajectory of the abrasive grain is visualized in Figure 3.

As illustrated in Figure 3, the abrasive grain’s trajectory exhibits an elliptical pattern under longitudinal–torsional composite vibration. Furthermore, within the *x-y* plane, the sinusoidal movement suggests periodic contact and separation between the abrasive particle and the workpiece along the torsional axis. During torsional vibration, the abrasive particles may move in the same or opposite direction as the primary motion. When the torsional vibration is synchronized with the spindle rotation, this motion increases the effective cutting speed and promotes material removal. In contrast, when the motion is in the opposite direction, the interaction between the abrasive grain and the workpiece shifts from cutting to a more friction-dominated process, which decreases the cutting force and enhances chip evacuation.

Furthermore, intermittent contact between abrasive grain and workpiece alters their interaction with materials. During the separation stages of contact cycles, empty cuts by abrasive grain occur on materials, reducing abrasion dullness. In contrast, during contact stages of cycles where acceleration is high for abrasive particles, concentrated energy is utilized for impact processing, resulting in easier breaking and removal of material fragments. Simultaneously, the separation processing stage allows cooling fluid to enter the cutting area more easily, ensuring thorough lubrication and cooling of abrasive particles while preventing adhesion between them and chips, thereby avoiding the secondary scratch phenomenon.

The velocity components of a single abrasive grain can be obtained by taking the derivative of Equation (1), as shown below:

(2) $$\left\{\begin{array}{l} v_{x}=\frac{\pi nR_{o}}{30}\cos\left(\frac{\pi n}{30}t\right)+2\pi fA_{T}\cos\left(2\pi ft\right)\cos\alpha +f_{r}\\ v_{y}=-\frac{\pi nR_{o}}{30}\sin\left(\frac{\pi n}{30}t\right)+2\pi fA_{T}\cos\left(2\pi ft\right)\sin\alpha \\ v_{z}=2\pi fA_{L}\cos\left(2\pi ft+\theta \right) \end{array}\right.$$

The distance traveled by an individual abrasive grain during one ultrasonic vibration period (*T* = 1/*f*) can be formulated as follows:

(3) $$\begin{array}{cc} L & =\int_{0}^{T}\sqrt{{v_{x}}^{2}+{v_{y}}^{2}+{v_{z}}^{2}}dt\\ & =\int_{0}^{1/f}\sqrt{{\left(\frac{\pi nR_{o}}{30}\right)}^{2}+\frac{4\pi^{2}fnR_{o}A_{T}}{30}\cos\left(2\pi ft\right)\cos\left(\frac{\pi n}{30}t+\alpha \right)+{\left(2\pi fA_{T}\cos\left(2\pi ft\right)\right)}^{2}+{\left(2\pi fA_{L}\cos\left(2\pi ft+\theta \right)\right)}^{2}}dt \end{array}$$

According to Equation (3) and Figure 3, LTC-RUM’s single abrasive cutting path exceeds that of CON-M, thereby enhancing material removal efficiency.

### 2.2. Material Removal Mechanism by a Single Abrasive Grain

**Figure 4:**
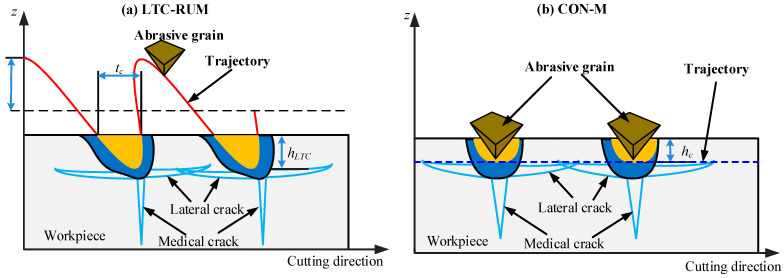
Cutting mechanism of the single abrasive grain in LTC-RUM (**a**) and CON m (**b**).

The effect of the velocity of an individual abrasive grain on the material removal mechanism in LTC-RUM is considered minimal. Figure 4 presents the cutting mechanism of an abrasive particle during LTC-RUM and CON-M, as depicted in Figure 4a, where the particle’s action can be likened to a series of indentations performed by Vickers indenters. Upon initial contact with the workpiece, the material undergoes elastic–plastic deformation. Once the indentation depth surpasses a certain threshold, median cracks begin to form. As the abrasive grain retracts, residual tensile stresses develop, which in turn trigger the formation of lateral cracks. During the subsequent engagement of the grain with the workpiece, these pre-formed lateral cracks hinder the further growth of median cracks and cause them to change direction. This interaction results in smaller indentation pits, reduced surface roughness, and an overall improvement in surface quality, as illustrated in Figure 4b. Unlike LTC-RUM, where the abrasive grain intermittently contacts the workpiece, continuous contact occurs in CON-M. As a result, the lateral cracks are unable to impede the progression of median cracks, leading to less effective grinding performance.

Based on the research results of Wang et al. [28], the contact time between a single abrasive grain and the workpiece within one vibration cycle during LTC-RUM can be calculated using the following expression:

(4) $$t_{\mathrm{LTC}}=\frac{\zeta }{\pi f}\left(\frac{\pi }{2}-\arcsin(1-\frac{h_{\mathrm{LTC}}}{A_{L}})\right)$$

where *h*_LTC_ denotes the penetration depth of the abrasive grains in the workpiece during LTC-RUM, *ζ* is determined by a proportional coefficient within the range of 0.5 < *ζ* < 1 [21].

Using the following approximation,

(5) $$\frac{\pi }{2}-\arcsin(1-\frac{h_{\mathrm{LTC}}}{A_{L}})\approx \sqrt{2h_{\mathrm{LTC}}/A_{L}}$$

Equation (5) can be simplified to the following form:

(6) $$t_{\mathrm{LTC}}\approx \frac{\zeta \sqrt{2}}{\pi f}{\left(\frac{h_{\mathrm{LTC}}}{A_{L}}\right)}^{\frac{1}{2}}$$

The abrasive grain in CON-M maintains continuous contact with the workpiece, as illustrated in Figure 4b. Consequently, the contact duration in LTC-RUM is significantly shorter than that of *t*_CON_ in CON-M.

Based on the research results of Jiao [29], the maximum force applied by an individual abrasive grain to the workpiece material during the CON-M process can be represented mathematically in the following manner:

(7) $$F_{m,\mathrm{CON}}^{\prime }=\frac{1}{2}{\mathrm{\lambda h}}_{\mathrm{CON}}^{2}\tan^{2}{\mathrm{\varphi H}}_{V}$$

where *λ* represents the geometric factor, *φ* denotes the vertical semi-angle of the indenter, and *H*_V_ stands for the microhardness of the workpiece material.

The material removal is presumed to be carried out by all active abrasive grains, each with a uniform penetration depth of *h*_CON_, and the maximum impact force *F*_*m*,CON_ can be calculated using the following expression:

(8) $$F_{m,\mathrm{CON}}=\frac{1}{2}{\mathrm{\lambda Nh}}_{\mathrm{CON}}^{2}\tan^{2}{\mathrm{\varphi H}}_{V}$$

where *N* represents the effective number of abrasive grains.

An average cutting force per vibration cycle of an approximate triangular waveform can be derived as follows:

(9) $$F_{c,\mathrm{CON}}T=\frac{1}{2}F_{m,\mathrm{CON}}t$$

Substituting Equation (8) into Equation (9) yields

(10) $$F_{c,\mathrm{CON}}=\frac{1}{4}{\mathrm{\lambda Nh}}_{\mathrm{CON}}^{2}\tan^{2}{\mathrm{\varphi H}}_{V}$$

By incorporating Equations (6) and (8) into Equation (9), the cutting force in LTC-RUM can be formulated as described in the following expression, focusing on the modeling procedure of the cutting force *F_c_*_,CON_:

(11) $$F_{c,\mathrm{LTC}}=\frac{\sqrt{2}\mathrm{\zeta N\lambda}}{4\pi \sqrt{A_{L}}}h_{\mathrm{LTC}}^{\frac{5}{2}}\tan^{2}{\mathrm{\varphi H}}_{V}$$

The cutting force in both CON-M and LTC-RUM is influenced by the indentation depth, as shown in Equations (10) and (11), respectively. From these equations, the following relationship can be established:

(12) $$\frac{F_{c,\mathrm{LTC}}}{F_{c,C\mathrm{ON}}}=\frac{\sqrt{2}{\mathrm{\zeta h}}_{\mathrm{LTC}}^{\frac{5}{2}}}{\pi \sqrt{A_{L}}h_{\mathrm{CON}}^{2}}\approx \frac{\sqrt{2}\zeta }{\pi }{\left(\frac{h_{\mathrm{LTC}}}{h_{\mathrm{CON}}}\right)}^{2}=k{\left(\frac{h_{\mathrm{LTC}}}{h_{\mathrm{CON}}}\right)}^{2}$$

where $k=\sqrt{2}\zeta /\pi$, 0.22 < *k* < 0.45.

To explore the fracture behavior of hard and brittle materials under applied loads, the micro-indentation testing approach was utilized to determine the critical cutting depth, based on indentation fracture mechanics, as expressed in the following equation [30]:

(13) $$h_{c}=\frac{EG}{H_{V}^{2}}$$

where *E* and *G* denote the material’s elastic modulus and fracture energy, respectively.

When the material fractures under static conditions due to slow loading, there are

(14) $$G\propto \frac{K_{c}^{2}}{H_{V}}$$

where *K_c_* represents the static fracture toughness of the material.

Substituting Equation (13) into (14), the following equation can be obtained:

(15) $$h_{c}\propto \left(\frac{E}{H_{V}}\right){\left(\frac{K_{c}}{H_{V}}\right)}^{2}$$

When the number of cracks on the surface of the hard and brittle material is below 10%, taking into account the influence of diamond indenter head geometry on the critical cutting depth, the following requirement on the critical cutting depth for the material should be met [31]:

(16) $$h_{c}=\frac{\chi }{\tan\varphi }{\left(\frac{2\psi }{\beta }\right)}^{\frac{1}{2}}{\left(\frac{K_{c}}{H_{V}}\right)}^{2}$$

where *χ* is a dimensionless parameter that depends on the processing parameters and machine performance, *ψ* is the integrated coefficient, and *β* is the factor related to the indenter head geometry.

Based on the indentation test findings reported by George et al. [32] regarding the fracture behavior of hard and brittle materials under equilibrium loading conditions, the following empirical formula was established:

(17) $$P_{c}{=\mathrm{\psi K}}_{c}{\left(\frac{K_{c}}{H_{V}}\right)}^{3}$$

where *P_c_* is the critical pressure load.

The relationship between the critical cutting depth *h_c_* of brittle plastic and the critical compression load *P_c_* can be described by Equations (7) and (8):

(18) $$h_{c}=\frac{\chi }{\tan\varphi }{\left(\frac{2P_{c}}{\beta H_{V}}\right)}^{\frac{1}{2}}$$

During the cutting process, the actual cutting motion is a dynamic result of various motion modes, such as rotational and feed motions. However, the static fracture toughness *K_c_* in Equation (14) is obtained under static conditions. Therefore, the calculated critical cutting depth of brittle plastic transformation *h_c_* only applies to cutting under static conditions. According to Kalthoff et al. [33], further research on dynamic fracture toughness under impact loads reveals that for ceramic hard brittle materials, their dynamic fracture toughness decreases with increasing loading rate, which significantly deviates from studying dynamic crack initiation rules based solely on static fracture toughness and fails to accurately reflect material behavior under impact loads. Considering the significant influence of cutting processing at the grain–workpiece contact interface, it becomes crucial to account for dynamic impact load effects in order to understand the cutting process of hard and brittle materials properly. Due to multiple motion patterns involved in the cutting process, along with factors like grain geometry, machine performance, cooling conditions, etc., it becomes highly complex; moreover, since the direction of dynamic impact load applied by the tool onto the workpiece surface may not be perpendicular and changes over time during machining operations, obtaining accurate results becomes challenging. Henceforth, only *h_c_* can be used qualitatively to analyze the brittle plastic transition properties of hard and brittle materials.

The utilization of ultrasonic vibration in cutting processes results in a reduction in the fracture toughness of the material compared to conventional cutting methods, facilitating the generation of cutting cracks and filings. This phenomenon contributes to a decrease in cutting force and an improvement in material surface quality [34]. In accordance with Equation (18), ultrasonic vibration can enhance the critical cutting depth when compared to conventional cutting techniques under identical cutting forces. Therefore, the relationship between penetration depths *h*_LTC_ and *h*_CON_ can be expressed as *h*_LTC_/*h*_CON_ > 1. At the same time, *F_c_*,_LTC_/*F_c_*,_CON_ < 1 according to Equation (12). These findings indicate that during LTC-RUM processing, there is potential for reducing the cutting force compared to CON-M.

## 3. Experimental Setup and Methodology

### 3.1. Experimental Setup

**Figure 5:**
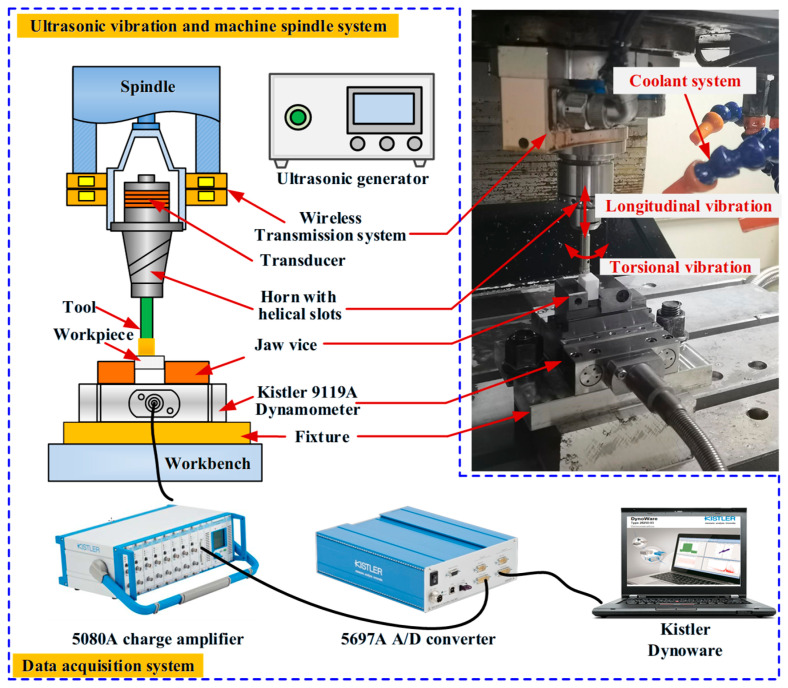
Experimental setup for LTC-RUM and CON-M.

The experiments for LTC-RUM and CON-M were carried out on a CNC machining center (HenFux-HFM700 L, Henfux Technology Group, Shenzhen, China). A custom ultrasonic vibration system, mainly composed of a transducer and a helical-slotted horn, was employed. The transducer transformed electrical energy into mechanical oscillations, which were then transmitted along the longitudinal axis. The horn served to magnify these longitudinal vibrations and partially convert them into torsional vibrations via its helical grooves. Finally, the combined longitudinal–torsional vibrations were delivered to the tool’s end surface. The underlying theory of this vibration system has been detailed in prior studies [19]. A diagram illustrating the experimental configuration and the setup structure is presented in Figure 5.

As illustrated in Figure 5, the ultrasonic vibration unit was mounted onto the spindle, with the required electrical oscillations supplied by an ultrasonic power source capable of delivering up to 500 W. The cutting force measurement system primarily included a Kistler 9119AA dynamometer, a charge amplifier (5080A), a data acquisition device (5697A), and Kistler DynoWare software for data collection. A dedicated fixture was used to mount and stabilize the dynamometer, which was then linked to the jaw chuck for enhanced rigidity. The workpiece was securely clamped by the jaw chuck throughout the machining process. The dynamometer’s electrical output was first amplified via the charge amplifier and then transmitted to the data recorder (5697A). The recorded signals were subsequently stored and visualized on a computer using Kistler DynoWare software. Considering that the ultrasonic frequency was considerably higher than the natural frequency of the dynamometer, average cutting force values were used to assess the machining performance. Additionally, a coolant system was implemented throughout the experiment to ensure proper thermal management during machining. The cutting force in the *z*-axis direction was specifically monitored during the tests.

**Figure 6:**
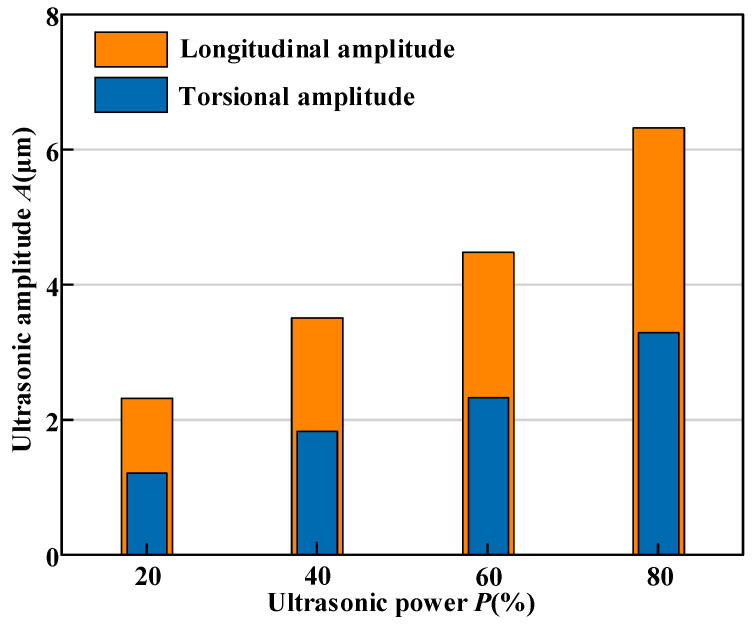
Relationship between ultrasonic power and the corresponding variation in ultrasonic amplitude.

Within the ultrasonic vibration setup, an ultrasonic power supply with a maximum output of 500 W transforms alternating current into high-frequency electrical oscillations, which are then transmitted to the transducer to be converted into high-frequency mechanical vibrations. A horn is employed to boost these vibrations to the desired levels. The horn is designed with helical grooves that facilitate the partial conversion of longitudinal vibration into torsional vibration. During the machining process, ultrasonic power is used to track changes in vibration amplitude. A laser displacement sensor (LKG10, KEYENCE, Osaka, Japan) was applied to measure both the torsional (*A*_T_) and longitudinal (*A*_L_) vibration amplitudes at the tool’s output surface. The average ratio of torsional to longitudinal amplitude (*A*_T_/*A*_L_) was determined to be 0.52, as shown in Figure 6.

**Figure 7:**
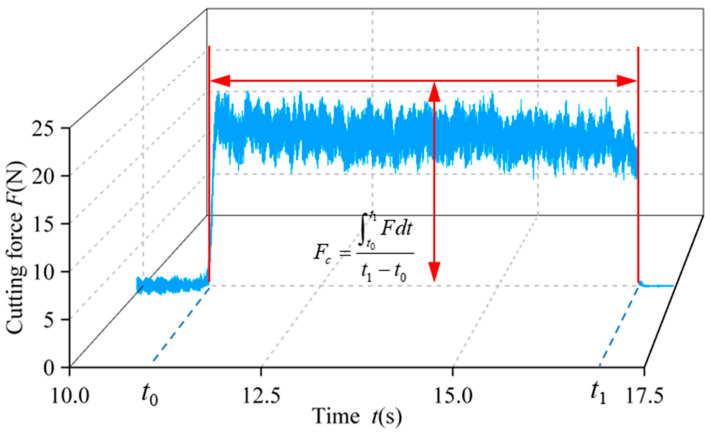
Cutting force evolution.

The diamond core tool was attached to the horn using an ER16 tapered connector, and the workpiece was firmly held in place by a jaw chuck mounted on a Kistler 9119AA dynamometer. The measured cutting force variation during the experiment is displayed in Figure 7. The average cutting force, denoted as *F_c_*, was determined using the following formula:

(19) $$F_{c}=\frac{\int_{t_{0}}^{t_{1}}\mathrm{Fdt}}{t_{1}-t_{0}}$$

where *F* represents the time-dependent drilling force measured during the process, while *t*_0_ and *t*_1_ indicate the moments when the tool initiated and finished the drilling operation, respectively.

### 3.2. Design of Experiment

The workpiece material used in this study was zirconia ceramics, and its mechanical properties are presented in Table 1. All workpiece specimens were pre-fabricated with the dimensions of 16 × 16 × 10 mm.

The diamond core tool used in the experiment was manufactured by the Zhengzhou Research Institute for Abrasives & Grinding Co., Ltd., Zhengzhou, China, and its specific parameters are listed in Table 2.

During the experiment, the tool’s resonant frequency was recorded as 34.89 kHz at a tuning length of 30 mm. While the ultrasonic frequency was kept constant throughout the testing, adjustments were made to parameters such as spindle speed, feed rate, cutting depth, and ultrasonic amplitude. The complete set of machining parameters is summarized in Table 3. Both the LTC-RUM and CON-M processes were performed with the capability to activate or deactivate the ultrasonic generator. The results presented here are the average values obtained from three repeated trials for each experimental condition.

The surface roughness was measured employing a white light interferometer (NPFLEX, Bruker, Billerica, MA, USA). To assess the three-dimensional surface roughness (*Sa*) following machining, five uniformly distributed measurement points were chosen. The surface morphology of the workpiece was examined using a scanning electron microscope (Merlin Compact, ZEISS, Oberkochen, Germany).

## 4. Results and Discussion

### 4.1. Cutting Force

**Figure 8:**
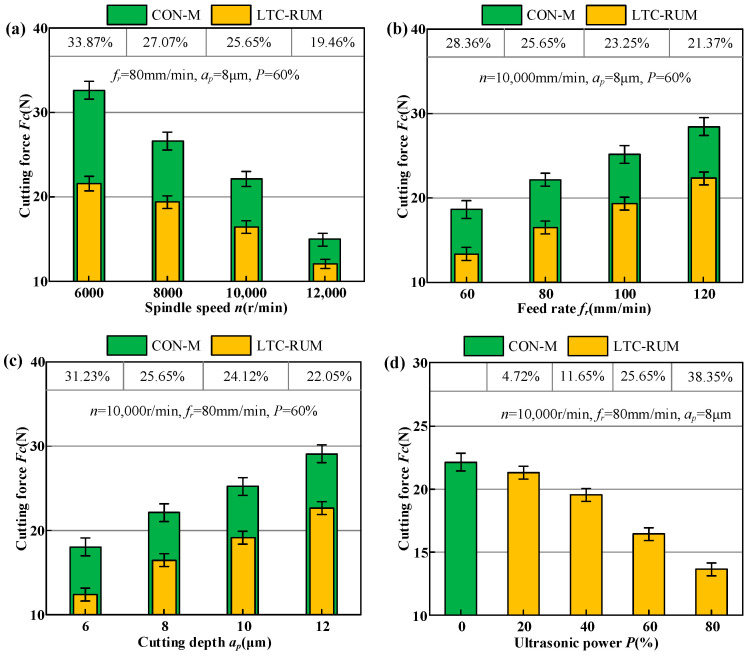
The impact of different parameters on cutting force: (**a**) spindle speed, (**b**) feed rate, (**c**) cutting depth, and (**d**) ultrasonic power.

Examining how spindle speed, feed rate, cutting depth, and ultrasonic power affect the cutting force in both machining techniques can help optimize the selection of more efficient methods and parameters for processing zirconia ceramics using CON-M and LTC-RUM. Figure 8 presents the influence of these machining parameters on cutting force, with the cutting force reduction ratio of LTC-RUM compared to CON-M shown at the top.

As shown in Figure 8a, the cutting force *F_c_* consistently decreased with increasing spindle speed across all tested conditions. This trend can be explained by the fact that a higher spindle speed increases the cutting path length per abrasive grain, assuming constant feed rate and cutting depth. This leads to a greater number of active abrasive grains participating in the cutting process, which in turn reduces the average contact area between individual grains and the workpiece, resulting in lower cutting forces. Compared to conventional machining (CON-M), the maximum reduction in cutting force reached 33.87%. Figure 8b,c demonstrate that the cutting force increased with higher feed rates and deeper cuts. When the feed rate was increased, fewer abrasive grains were dynamically engaged in the grinding zone, which led to a rise in cutting force. In comparison to CON-M, the maximum reductions in cutting force under these conditions were 28.36% and 31.23%, respectively. An increase in cutting depth also resulted in greater resistance to abrasive grain penetration into the workpiece, thereby increasing the grinding force. As illustrated in Figure 8d, the application of ultrasonic power contributed to a reduction in cutting force. The enhanced ultrasonic amplitude extended the cutting length of each abrasive grain while maintaining a constant number of active grains and material removal rate. However, to keep the material removal rate unchanged, the average cutting depth per abrasive grain must be reduced, as previously confirmed by Cong et al. [35]. As a result, higher ultrasonic power levels corresponded to lower cutting forces, with a maximum reduction of 38.35% observed in this study.

### 4.2. Surface Roughness

**Figure 9:**
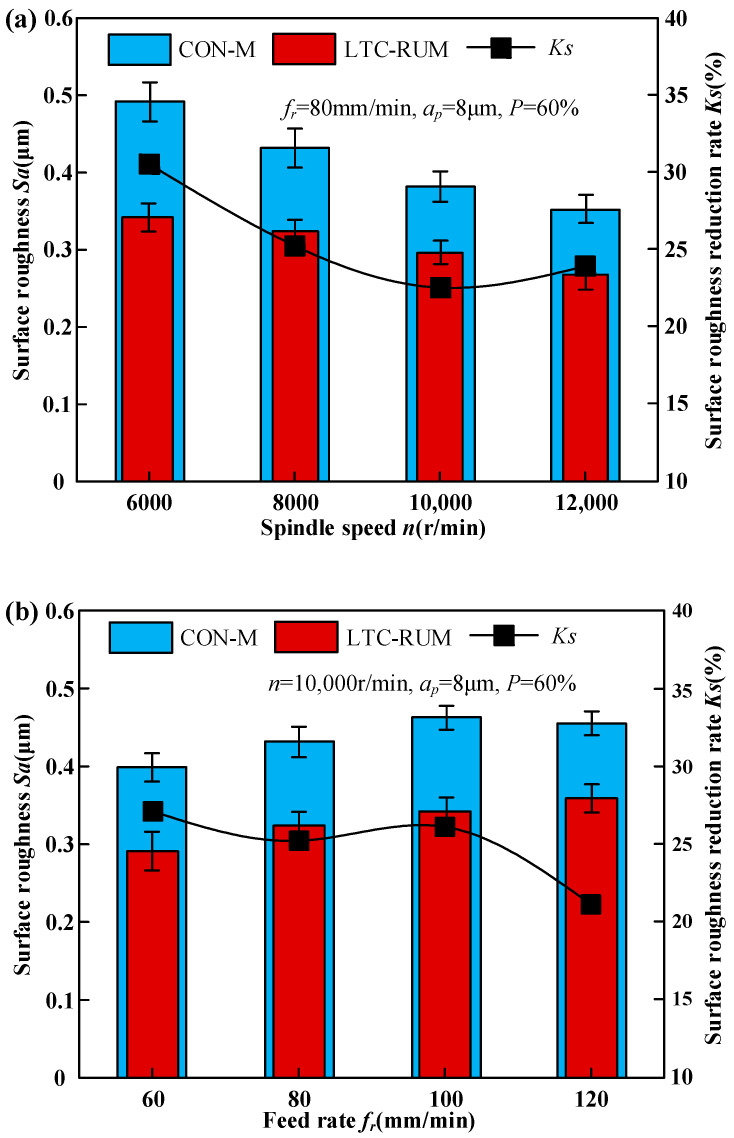

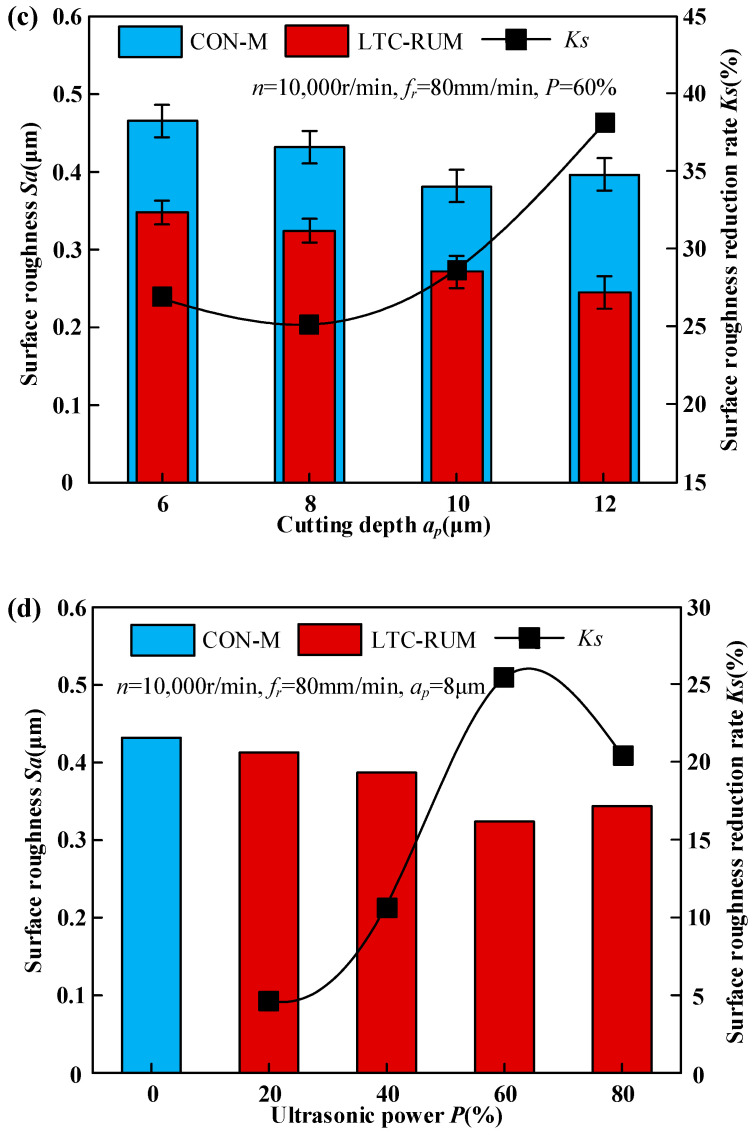
The impact of processing parameters on surface roughness: (**a**) spindle speed, (**b**) feed rate, (**c**) cutting depth, and (**d**) ultrasonic power.

Figure 9 presents the influence of machining parameters on surface roughness. To evaluate the effectiveness of longitudinal–torsional composite vibration in reducing surface roughness relative to conventional machining, the index *Ks* was introduced. This index can be formulated as follows:

(20) $$Ks=\frac{Sa_{\mathrm{CON}}-Sa_{\mathrm{LTC}}}{Sa_{\mathrm{CON}}}$$

where $Sa_{\mathrm{CON}}$ and $Sa_{\mathrm{LTC}}$ denote the surface roughness values obtained from CON-M and LTC-RUM, respectively. A greater *Ks* value signifies a higher degree of surface roughness improvement.

As illustrated in Figure 9a, the surface roughness *Sa* decreased as the spindle speed increased. Under conditions of 80 mm/min feed rate, 8 μm cutting depth, and 60% ultrasonic power, the *Ks* value dropped from 30.5% to 22.5% when the spindle speed was raised from 6000 to 12,000 r/min. The rise in spindle speed led to a greater number of abrasive grains participating in the grinding process per unit time, which reduced the undeformed chip thickness per grain and consequently lowered the surface roughness. In the LTC-RUM process, the introduction of torsional vibration further elevated the grinding speed and reduced the undeformed chip thickness, resulting in fewer median cracks and a higher proportion of material removed through plastic deformation, thereby effectively reducing Sa. However, as spindle speed continued to increase, the intermittent contact between abrasive grains and the workpiece weakened, partially diminishing the advantages of ultrasonic vibration. This led to a reduction in the effectiveness of surface roughness improvement, as reflected by the declining *Ks* value.

Figure 9b demonstrates that during CON-M, the surface roughness *Sa* initially decreased and then increased with rising cutting depth, whereas in the LTC-RUM process, *Sa* continued to decrease. At a spindle speed of 10,000 r/min, a cutting depth of 8 μm, and ultrasonic power of 60%, the *Ks* value dropped from 27.1% to 21.1% as the feed rate increased from 60 mm/min to 120 mm/min. In the CON-M process, when the feed rate was below 100 mm/min, surface roughness first increased and then decreased due to the growing resistance between abrasive grains and the workpiece, which initially hindered efficient material removal. However, at feed rates above 100 mm/min, *Sa* slightly decreased. In contrast, during LTC-RUM, higher feed rates reduced the intermittent contact between abrasive particles and the workpiece, increasing the contact duration while decreasing the effective cutting time. This resulted in a higher material removal rate and increased friction between the abrasive grains and the workpiece material. Such conditions promoted crack propagation in brittle materials, leading to the removal of larger material fragments and more pronounced surface degradation.

The surface roughness exhibited an initial decrease followed by an increase with increasing cutting depth during CON-M, whereas it decreased consistently during LTC-RUM, as shown in Figure 9b. At a spindle speed of 10,000 r/min, a cutting depth of 8 μm, and an ultrasonic power of 60%, the value of *Ks* decreased from 27.1% to 21.1% as the feed rate increased from 60 mm/min to 120 mm/min. In the CON-M process, when the feed rate was below 100 mm/min, *Sa* initially increased and then decreased, with further increases in feed rate due to enhanced resistance between abrasive grain and workpiece hindering effective material removal. However, for feed rates exceeding 100 mm/min, *Sa* slightly decreased instead. Conversely, in the LTC-RUM process, higher feed rates weakened intermittent contact between abrasive particles and workpiece, resulting in increased contact time between abrasive grains and material but reduced effective cutting time. This led to an elevated material removal rate and relative friction between abrasive grain and material, which facilitated the expansion of cracks in brittle materials, causing the removal of larger pieces and leading to noticeable surface wear.

The surface roughness exhibited an initial increase followed by a decrease with increasing cutting depth during CON-M, as depicted in Figure 9c, whereas it increased during LTC-RUM. At a spindle speed of 10,000 r/min, a feed rate of 100 mm/min, and ultrasonic power of 60%, the value of *Ks* decreased from 38.1% to 25.3% as the cutting depth increased from 6 μm to 12 μm. In the CON-M process, when the cutting depth was below 10 μm, *Sa* initially decreased and then increased with increasing cutting depth. With further increases in cutting depth, the material removal mechanism transitioned from plastic removal to plastic–brittleness removal and eventually predominantly brittle removal. For small cutting depths, there were fewer grinding particles per unit area, resulting in larger values of *Sa*. However, at approximately 10 μm or 12 μm cutting depths, material removal occurred through plastic–brittle binding mode, leading to the lowest surface roughness *Sa* for both cases considered here. Notably, CON-M demonstrated a greater propensity for achieving minimum *Sa* values than LTC-RUM due to its ability to reach critical cutting depths more effectively and induce brittle zone grinding effects. The application of longitudinal–torsional coupled vibration facilitated shifting back the brittle–plastic transition depth in ceramic materials, thereby enabling easier realization of plastic zone machining processes. Consequently, LTC-RUM exhibited lower surface roughness than CON-M.

As shown in Figure 9d, the surface roughness *Sa* first decreased and then increased with rising ultrasonic power. The *Sa* value ranged from 4.4% to 24.8%, peaking at an ultrasonic power setting of 60%. At lower power levels, the energy delivered to the workpiece by the abrasive grains was inadequate, leading to weakened intermittent contact between the grains and the material surface. However, as ultrasonic power increased, this intermittent contact was strengthened, promoting more effective intermittent cutting and enhancing material removal through the formation of finer chips. Moreover, with higher ultrasonic power, the impact force of the abrasive grains on the workpiece surface increased, resulting in a greater cutting depth per grain. This larger cutting depth caused more material to be removed in a single interaction, leading to wider and deeper surface scratches, which in turn increased surface roughness. Nevertheless, the application of longitudinal–torsional coupled vibration enhanced the back-and-forth motion of the abrasive grains on the material surface. At the same time, under ultrasonic vibration, abrasive grains experienced more frequent non-cutting (empty cutting) phases, which helped reduce secondary surface damage.

### 4.3. Surface Morphology

**Figure 10:**
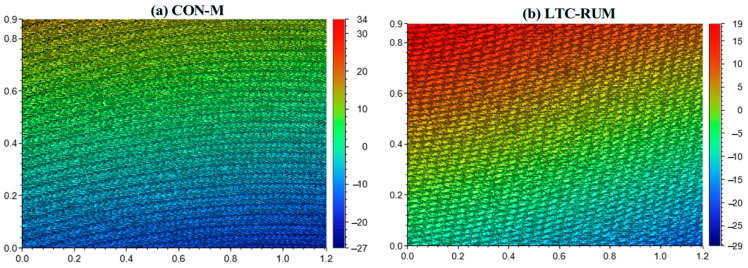
3D surface morphology (*n* = 10,000 r/min, *f_r_* = 80 mm/min, *a_p_* = 8 μm, *P* = 60%): (**a**) CON-M; (**b**) LTC-RUM.

The 3D surface morphology and microstructural characteristics of the machined surfaces were analyzed with specific emphasis on surface texture. The comparison between CON-M and LTC-RUM is presented in Figure 10, which highlights a notable enhancement in surface quality when using LTC-RUM over CON-M. As shown in Figure 10a, the conventional machining process produced clear grinding grooves and raised ridges on the surface. In contrast, Figure 10b reveals that the application of longitudinal–torsional coupled vibration promotes interaction between the abrasive grains and the workpiece, contributing to a significant improvement in surface finish during LTC-RUM.

**Figure 11:**
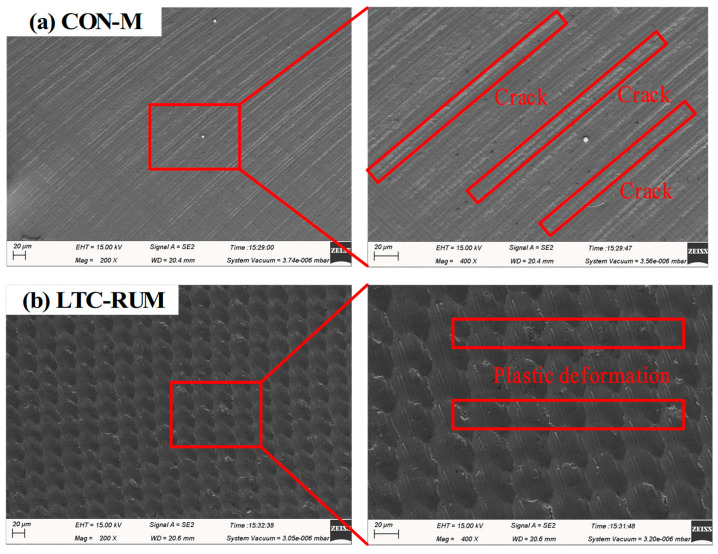
SEM images and their magnified fragments for CON-M (**a**) and LTC-RUM (**b**).

The SEM images were utilized to investigate the material removal mechanisms involved in both machining processes, as illustrated in Figure 11. As shown in Figure 11a, the CON-M process resulted in visible grain scratches on the surface, accompanied by the formation of multiple crystallographic fracture cracks. In contrast, under the influence of longitudinal–torsional coupled vibration in LTC-RUM (Figure 11b), overlapping abrasive grain paths were observed, along with pronounced plastic deformation features. A high-magnification SEM image from Figure 11b further revealed evidence of material tearing and localized deposition during this process. These observations indicate that the two methods operate through distinct material removal mechanisms, with LTC-RUM primarily relying on plastic deformation to remove material, which ultimately contributes to improved surface quality.

## 5. Conclusions

This research conducted a theoretical examination of the abrasive grain trajectories and material removal behavior in LTC-RUM applied to ZrO_2_ ceramics. Additionally, an experimental study was carried out to evaluate and compare the machining performance of LTC-RUM and CON-M in terms of cutting force, surface roughness, and surface topography. Based on the findings, the following conclusions were reached:

(1) A kinematic analysis of the motion paths of individual abrasive grains in both machining methods was carried out. The intermittent interaction between the abrasive grains and the workpiece in LTC-RUM altered the material removal mechanism, thereby enhancing the efficiency of plastic material removal.
(2) Both cutting force *F_c_* and surface roughness showed a decreasing trend with increased spindle speed and ultrasonic power, while they increased with higher feed rates and cutting depths in both LTC-RUM and CON-M. Experimental results demonstrated that LTC-RUM, when operated under appropriate machining conditions, including optimal spindle speed, feed rate, cutting depth, and ultrasonic power, achieved considerable reductions in cutting force and surface roughness compared to CON-M. Specifically, cutting force was reduced by 33.87% to 38.35%, and surface roughness improvement ranged from 24.8% to 38.1%.
(3) The material removal mechanisms in CON-M and LTC-RUM were found to differ significantly. The critical cutting depth in LTC-RUM was higher than that in CON-M, as material removal in CON-M primarily involved crack propagation, whereas LTC-RUM relied more on plastic deformation. The integration of longitudinal–torsional coupled vibration notably enhanced the surface microstructural quality of the machined ZrO_2_ ceramics.

## Figures and Tables

**Table 1:** Mechanical properties of the zirconia ceramics.

Property	Elastic Modulus (GPa)	Hardness (GPa)	Fracture Toughness (MPa m^1/2^)	Density (g/cm^3^)	Poisson’s Ratio
Value	210	12	6	6.05	0.3

**Table 2:** Core tool parameters.

Mesh Size	Abrasive Particle Size *d*_a_ (mm)	Abrasive Concentration *C*_a_	Outer Diameter *d*_o_ (mm)	Inner Diameter *d*_i_ (mm)
#80–100	0.162	100	8.07	6.80

**Table 3:** Machining parameters during LTC-RUM and CON-M.

Variable	Values
Spindle speed *n* (r/min)	6000, 8000, 10,000, 12,000
Cutting depth *a_p_* (μm)	6, 8, 10, 12
Feed rate *f_r_* (mm/min)	60, 80, 100, 120
Ultrasonic power *P* (%)	0, 20, 40, 60, 80

## Data Availability

The original contributions presented in this study are included in the article.
